# Schizophrenia and oxidative stress from the perspective of bibliometric analysis

**DOI:** 10.3389/fpsyt.2023.1145409

**Published:** 2023-02-27

**Authors:** Meng-Yi Chen, Qinge Zhang, Yu-Fei Liu, Wan-Ying Zheng, Tong Leong Si, Zhaohui Su, Teris Cheung, Todd Jackson, Xiao-Hong Li, Yu-Tao Xiang

**Affiliations:** ^1^Unit of Psychiatry, Department of Public Health and Medicinal Administration, and Institute of Translational Medicine, Faculty of Health Sciences, University of Macau, Macau, Macao SAR, China; ^2^Centre for Cognitive and Brain Sciences, University of Macau, Macau, Macao SAR, China; ^3^The National Clinical Research Center for Mental Disorders and Beijing Key Laboratory of Mental Disorders, Beijing Anding Hospital & the Advanced Innovation Center for Human Brain Protection, Capital Medical University, Beijing, China; ^4^School of Public Health, Southeast University, Nanjing, China; ^5^School of Nursing, Hong Kong Polytechnic University, Hong Kong, Hong Kong SAR, China; ^6^Department of Psychology, University of Macau, Macau, Macao SAR, China; ^7^Peking University Huilongguan Clinical Medical School, Beijing Huilongguan Hospital, Beijing, China

**Keywords:** schizophrenia, oxidative stress, bibliometric analysis, antioxidants, nitric oxide

## Abstract

**Background:**

A growing number of studies has implicated oxidative stress in the pathophysiology of psychiatric disorders including schizophrenia. The aim of this study was to explore the field of schizophrenia and oxidative stress-related research from a bibliometric perspective.

**Methods:**

All relevant publications on schizophrenia and oxidative stress were obtained from Web of Science Core Collection (WOSCC) database from its inception date to November 8, 2022. VOSviewer software was used to examine co-authorships and co-occurring keywords. R software was used to present the main characteristics of publications and cooperation frequency among countries. CiteSpace was used to investigate keywords with the strongest citation bursts.

**Results:**

A total of 3,510 publications on schizophrenia and oxidative stress were included. The United States had the largest number of publications (26.1%), and international collaborations. University of Melbourne was the most productive institution, while Schizophrenia Research was the most productive journal in this field. Apart from “schizophrenia” and “oxidative stress”, the terms “prefrontal cortex”, “brain” and “nitric oxide” were among the most frequently used keywords.

**Conclusions:**

In conclusion, research on the association between oxidative stress and schizophrenia has received growing attention in the academic literature that is expected to continue its upward trajectory during the next two decades. Existing research suggests there has been a transition from research focused on pathways to animal models, and subsequently to clinical applications. Intervention studies on oxidative stress and schizophrenia are likely to be an important focus of related work in the near future.

## 1. Introduction

Schizophrenia is a severe psychiatric disorder characterized by positive symptoms (e.g., hallucinations and delusions) and negative symptoms (e.g., affective blunting and avolition) as well as a range of deficits in emotional, motivational, social, and cognitive functioning (1). The lifetime prevalence of schizophrenia is around 1% and the corresponding global burden of disease is estimated to be approximately 13 million years of life lived with disability (2). Mechanisms underlying schizophrenia are complicated, though the role of oxidative stress has been gaining attention (3).

Oxidative stress is a phenomenon caused by an imbalance between production and accumulation of oxygen reactive species in cells and tissues and the ability of a biological system to detoxify these reactive products (4). A meta-analysis revealed that schizophrenia patients show abnormalities in a variety of oxidative stress-related peripheral blood biomarkers, independent of antipsychotic medications used and clinical status (5). Some studies have found that certain cellular antioxidants and redox regulators are reduced in cerebrospinal fluid (6) and striatum postmortem tissues (7) of patients with schizophrenia. Animal studies on glutamate cysteine ligase (GSH; a cellular nonprotein antioxidant)—deficient models have found changes in morphology, electrophysiology, and abnormal behaviors similar to those observed in schizophrenia patients (8–10). Genome-wide analyses have also found that certain schizophrenia susceptibility genes converge on interconnected pathways associated with oxidative stress (11).

Bibliometric analysis is a widely used approach for performing quantitative and qualitative analyses of publications in specific research fields (12). The approach is useful for illuminating various features of publications including countries, institutions and authors that have generated the most publications, international research collaborations, the most influential journals, recent research trends, and the evolution and developmental frontiers of a particular research field (13). To date, no bibliometric analysis has been published on research assessing relations between schizophrenia and oxidative stress. To fill this gap, we conducted a bibliometric analysis on schizophrenia and oxidative stress-related research.

## 2. Methods

### 2.1. Data acquisition and search strategy

Web of Science (WOS) is the most commonly used academic information resource databases for bibliometric analysis. As recommended previously (14), relevant literature was searched in the Science Citation Index Expanded (SCI-E) and Social Sciences Citation Index (SSCI), both of which are included in the Web of Science Core Collection (WOSCC) (15). In this study, a literature search was performed from dates of inception to November 8, 2022. Search terms are shown in the Supplementary material. No limitations were placed on publication language or type. Relevant articles were exported to “Plain text file” or “Tab delimited file” formats.

### 2.2. Data analysis

R (version 4.0.4) (16), VOSviewer (version 1.6.17) (17), CiteSpace Software (6.1.R2) (18), and an online bibliometrics platform (https://bibliometric.com/) were used for data analysis. The “Bibliometrix” (16) package of R was used for statistical calculation and mapping of the following domains: (1) characteristics of publications (e.g., authorship, source and citation); (2) annual publication number and average growth rate; (3) cutting-edge keywords, authors and journals; (4) collaboration frequency among countries.

VOSviewer is a program for visualizing bibliometric maps. In such visualizations, a node represents an item (i.e., the author or country), the size of the node reflects its importance (i.e., the frequency of co-authorship), and the color of the node indicates the cluster that includes the node as a member. Network connectivity represents collaborations between institutions or authors (19). Line width indicates the strength of the collaboration. In this study, VOSviewer was used to: (1) present collaboration analyses of authors and institutions and (2) perform the co-occurrence of keywords.

CiteSpace software was used to examine the dynamic visual evolution of bibliometric networks over time (20). In this study, CiteSpace was used to identify keywords with the strongest citations bursts during a particular period. Moreover, the online bibliometrics platform (https://bibliometric.com/) was used to visualize international collaborations between countries.

## 3. Results

### 3.1. Publication summary

A total of 3,510 articles on schizophrenia and oxidative stress were included, of which, 3,455 (98.4%) were published in English, 13 were published in Russian, 9 in German, 8 in French, 8 in Spanish, 6 in Turkish, 4 in Polish, 3 in Japanese, 3 in Portuguese, and 1 each in Chinese and Hungarian.

**Figure 1:**
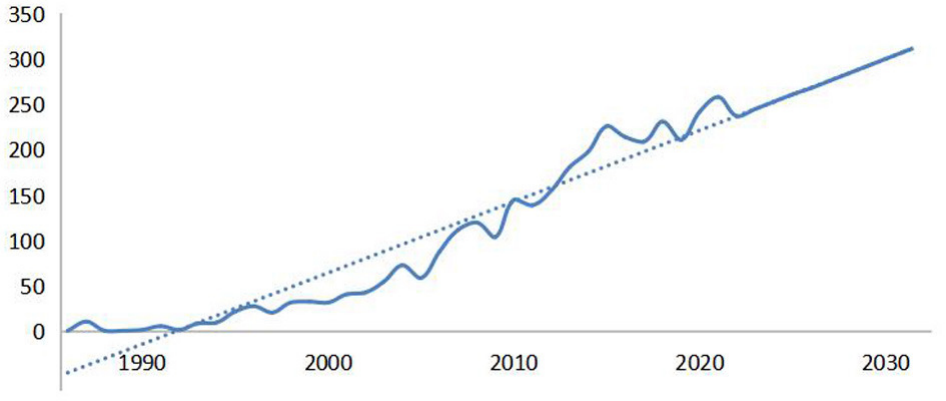
Number and trend of annual publication on schizophrenia and oxidative stress by year.

Figure 1 presents the number of publications on schizophrenia and oxidative stress by year. As a whole, the number of annual publication has been steadily increasing, with an annual growth rate of 1.89%. A regression analysis on the relationship between the number of articles and publication year (excluding 2022) generated an adequate fitting model for the annual publication trend (R^2^ = 0.9164). According to the prediction curve, the trend for annual publications on schizophrenia and oxidative stress shows an upward trajectory during the next 10 years; estimated annual numbers of papers are expected to increase to 276 by 2026 and to 316 by 2032.

### 3.2. Analysis of most productive countries and international collaborations

**Figure 2:**
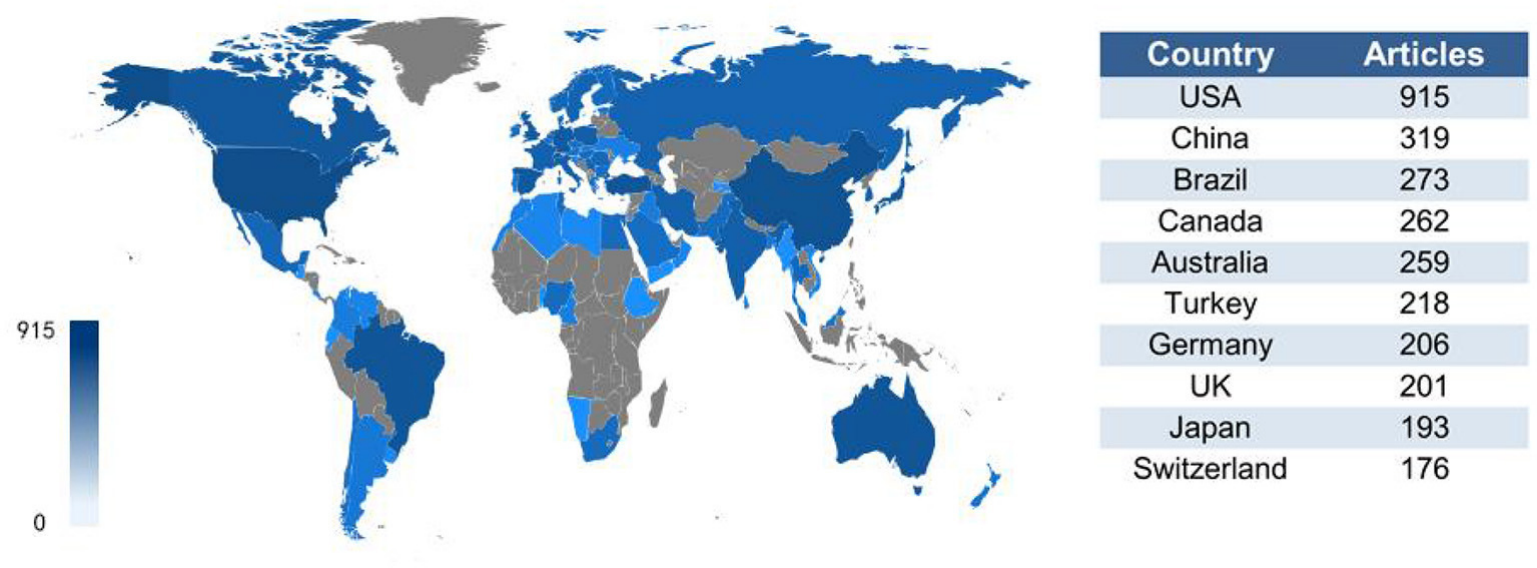
A visualization of publications by country based on publication counts.

Figure 2 presents the distribution of publications by country or region. The United States (U.S.) ranks first in the number of publications at 915 (26.1%), followed by China at 319 (9.1%) and Brazil at 273 (7.8%). Canada (7.5%), Australia (7.4%), Turkey (6.2%), Germany (5.9%), and the United Kingdom (U.K.) (5.7%) all had more than 200 publications in the research area of schizophrenia and oxidative stress.

Supplementary Figure 1 shows the pattern of collaborations between countries with at least 30 relevant collaborations. A majority of collaborations occurred between North American, European, Oceania, and East Asian countries. The most frequent international collaborations occurred between the U.S. and China (Frequency = 85), followed by Australia and Thailand (Frequency = 56), and between the U.S. and U.K. (Frequency = 52).

### 3.3. Analysis of the most productive institutions

**Figure 3:**
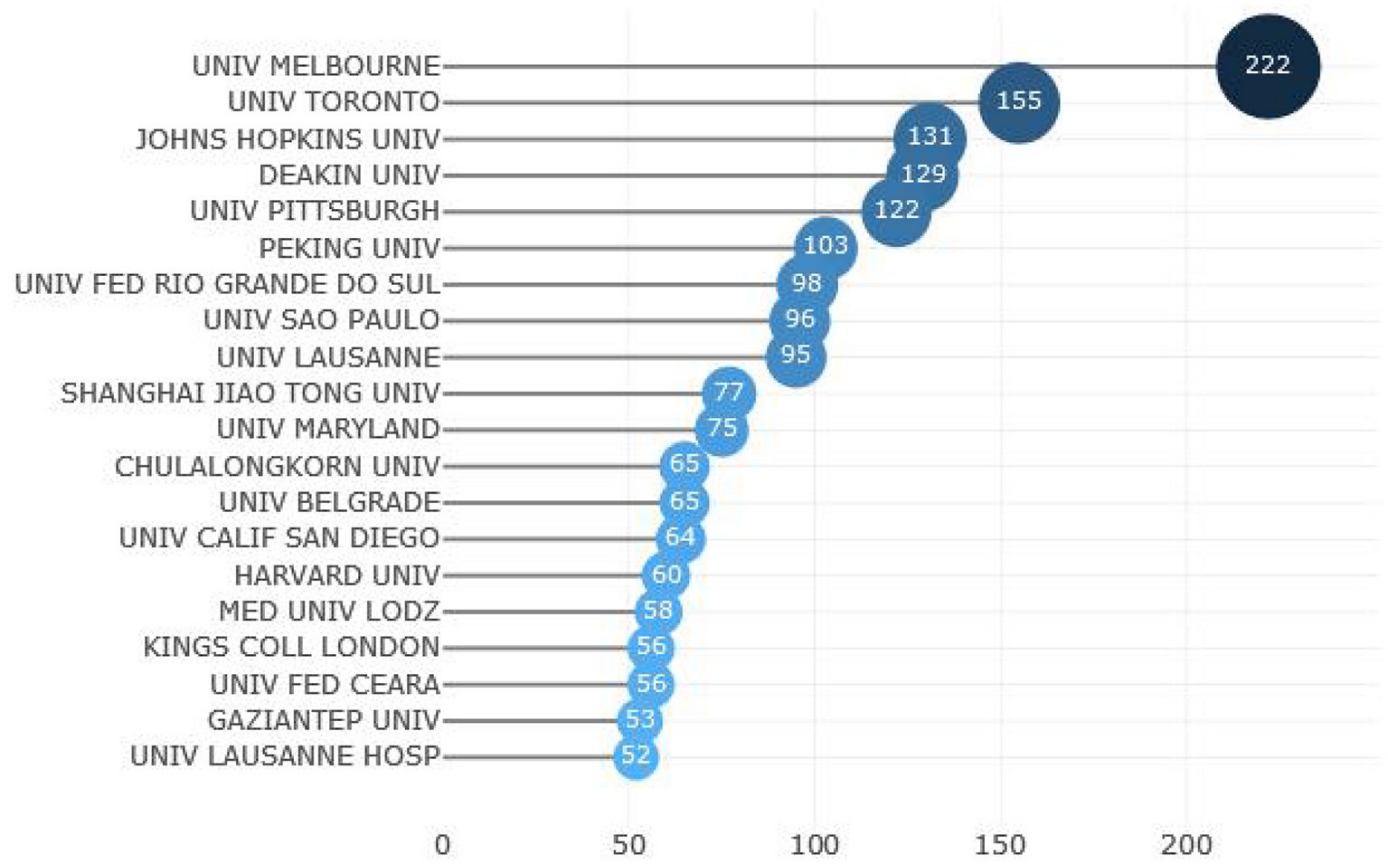
Top 20 affiliations with most publications in the field of schizophrenia and oxidative stress.

A total of 3,497 institutions contributed to research on schizophrenia and oxidative stress. Figure 3 shows the top 20 most productive institutions, with 6 from the U.S., 2 each from China, Australia, Brazil and Switzerland, 1 each from Canada, Poland, UK, Turkey, Serbia and Thailand. Of these, Melbourne University ranked first and published 222 articles. Institutional collaborations are shown in Supplementary Figure 2. With at least 25 publications in each institution, 40 institutions were included for analyses. Pittsburgh University and Georgia Medicine College in the U.S. pioneered institutional collaborations in the field of schizophrenia and oxidative stress (blue color). In contrast, more recently, Deakin University in Australia and Shanghai Jiao Tong University in China have initiated relevant research in this field (yellow color).

### 3.4. Analysis of the most productive authors

As shown in Table 1, 15,407 authors have published articles on schizophrenia and oxidative stress. Do, Kim Quang (Article:111, H-index:35, University of Lausanne), Berk, Michael (Article:94, H-index:42, Melbourne University) and Cuenod, Michel (Article:80, H-index:32, University of Lausanne) were authors with the most publications. Supplementary Figure 3 shows the overlap graph of collaborations between researchers. Using a minimum of 10 publications as the criterion for inclusion, 111 authors were included for analysis. Of those included, 66 had collaborated with other authors from this subset.

**Table 1:** Top 10 authors with the most publications in the field of schizophrenia and oxidative stress.

**Ranging**	**Author**	**Article**	**h-index**
1	Do, KQ	111	35
2	Berk, M	94	42
3	Cuenod, M	80	32
4	Maes, M	62	26
5	Zhang XY	56	25
6	Steullet, P	45	18
7	Yao, JK	45	23
8	Andreazza, AC	40	23
9	Sawa, A	39	21
10	Gama, CS	37	18

### 3.5. Analysis of the most productive journals

Articles on schizophrenia and oxidative stress have been published in 762 journals. Supplementary Table 1 shows the top 10 journals for publication frequency including impact factors and Journal Citation Reports categories (JCR-c); 40% of published papers appeared in Q1 journals while another 40% were published in Q2 journals. Regarding subject categories, certain journals are members of multiple categories. With this in mind, 60% of the papers were published in Psychiatry, 30% in Clinical Neurology, 20% in Neuroscience, and 10% in Biochemistry and Molecular Biology. In terms of publisher location, 80% were based in Europe and the other 20% were based in North America.

### 3.6. Analysis of publication trends

#### 3.6.1. Most cited articles

Citation analysis can reveal the influence of publications in a specific research field (21). Supplementary Table 2 lists the top 10 most-cited articles, each of which generated at least 400 citations. Among these, five are review articles and the other five are regular articles. The most highly cited paper was a review entitled “Glutamate uptake” that was published in 2001 and had 3,489 citations (22).

#### 3.6.2. Analysis of keywords

**Figure 4:**
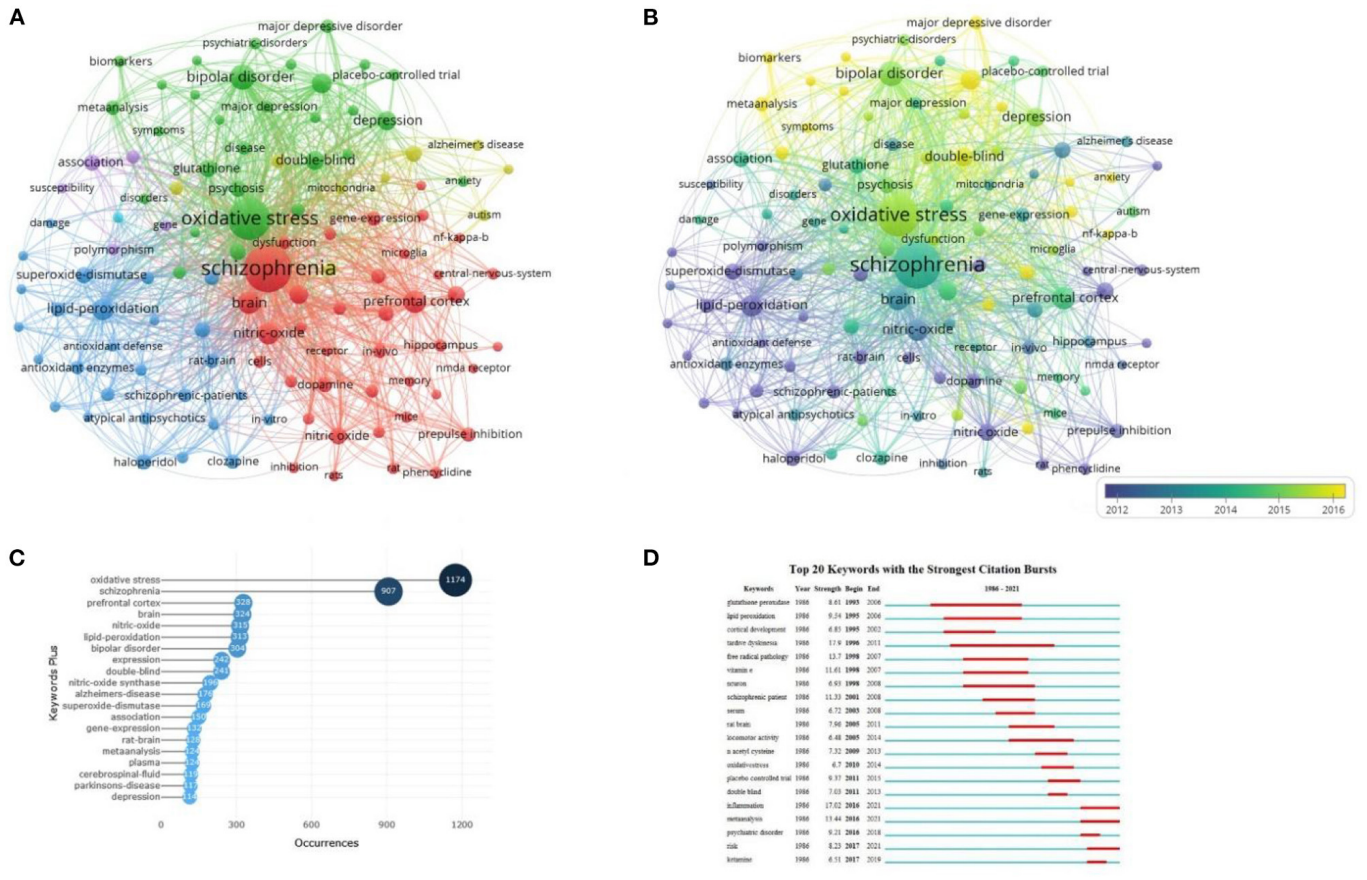
Analysis of the research hotspots on schizophrenia and oxidative stress. **(A)** Network visualization of all keywords. **(B)** Overlay visualization of all keywords. **(C)** Top 20 most frequent keywords. **(D)** Visualization map of top 20 keywords with the strongest citations bursts.

A total of 11,548 keywords were obtained from the included publications. Using the criterion of at least 50 occurrences, 111 keywords were included for analyses (Figure 4A). The size of nodes in the figure reflects the frequency of keywords, and the distance between nodes reflects the strength of the association between keywords. Keywords that are close to each other are grouped into the same cluster. Cluster 1 focused on schizophrenia, associated mechanism hypotheses and biomarkers, including “schizophrenia”, “dopamine”, “prefrontal cortex” and other keywords. Cluster 2 reflected other psychiatric disorders and clinical syndromes associated with oxidative stress such as “bipolar disorder”, “major depressive disorder” and “metabolic syndrome”. Cluster 3 focused on antipsychotics and relevant oxidative stress processes and included terms such as “haloperidol”, “clozapine” and “lipid-peroxidation”. Cluster 4 was related to neuropsychiatric disorders that may associated with oxidative stress such as “Alzheimer's disease” and “autism”. Cluster 5 was associated with epidemiological and genetic features of schizophrenia and featured keywords including “susceptibility” and “polymorphism”.

Figure 4B shows the overlay visualization of included keywords. Keywords that appeared earlier are marked in blue (e.g., “lipid-peroxidation”, “superoxide-dismutase”, “rat-brain”, “nitric oxide”, and “prepulse inhibition”) while those that appeared more recently are marked in yellow (e.g., “biomarker”, “major depressive disorder”, “double-blind”, “anxiety”, and “inflammation”). The top 20 most frequently occurring keywords are shown in Figure 4C. As expected, the keyword, “oxidative stress” was used most often (frequency = 1,174), followed by “schizophrenia” (*N* = 907), and “prefrontal cortex” (*N* = 328).

Figure 4D shows the top 20 keywords with the strongest citation bursts in schizophrenia and oxidative stress during the past 25 years. The length of the red line indicates the duration of relevant research. The keywords, “tardive dyskinesia” (1996–2011), “glutathione peroxidase” (1993–2006) and “lipid peroxidation” (1995–2006) had the longest previous citation bursts. More recently, the keywords, “inflammation” (2016–2021), “meta-analysis” (2016–2021), and “risk” (2017–2021) have generated citation bursts reflecting current and possible future trends in this field.

## 4. Discussion

This bibliometric analysis study examined trends in research on schizophrenia and oxidative stress. Analyses indicated publications on schizophrenia and oxidative stress have shown a steady increase during the past several decades in line with findings of previous “stand-alone” bibliometric studies on schizophrenia research (23) and oxidative stress and chemobrain (24). Advances in laboratory techniques and the discovery of various oxidative stress related-pathophysiological processes have likely facilitated the proliferation of research in this field (25). Overall, more than 98% of the included articles were published in English language outlets; this finding may be a partial function of using the WOSCC database which typically includes literature published in English.

This bibliometric analysis indicated that, to date, the U.S. has generated more publications than other countries on schizophrenia and oxidative stress in line with similar findings from bibliometric analyses conducted in fields of Psychiatry (26), Neuroscience (27), and Molecular Biology (28). This finding could reflect comparatively high investments in medical research in the U.S. as well as the country's high relative standing at economic academic, and health-care expenditure levels (29–31). Most international collaborations in the field have occurred between North American, European, Oceania, and East Asian countries. Country ranks in international collaborations greatly overlapped between the most research productive countries, suggesting that international academic collaborations may foster research outputs. Consequently, international collaborations should be encouraged in the future.

In terms of institutions, the U.S., China, Australia, Brazil and Switzerland each had at least two institutions among the top 20 most research prolific institutions. The U.S. had the most institutions (*N* = 6) in this subset, while the top two most productive institutions were in Australia (University of Melbourne, Article: 222) and Canada (University of Toronto, Article: 155), respectively. For authors, Do, Kim Quang (University of Lausanne, Article:111) from Switzerland was the most productive author, while Berk, Michael (Melbourne University, H-index:42) from Australia was the author with the most h-index article. From a global perspective, scientific research collaborations between institutions and authors is greatly influenced by region (32). Data from this paper suggest Western countries and institutions have been the main centers for research on schizophrenia and oxidative stress to date. Considering that collaboration is an effective way to promote the diversity of perspectives as well as the quantity and quality of publications, institutions and researchers from different regions should strengthen opportunities for cooperation and communication to advance the field further (14).

Regarding publication outlets for the field of schizophrenia and oxidative stress, eight of the top 10 most productive journals originated in the U.K. (*N* = 4) or European countries of the Netherlands (*N* = 3) or Germany (*N* = 1); the two remaining top 10 journals originated in the U.S. In contrast, no active publishers were located in China, Brazil, or Canada, although these were among the most productive countries. Schizophrenia Research was the most productive journal, consistent with findings of other bibliometric analyses on schizophrenia related cognitive behavioral therapy (33) and Magnetic Resonance Imaging research (34). Moreover, the top 10 most cited papers were all published before 2014 and focused primarily upon oxidative stress-related changes in schizophrenia patients (35–37), use of antioxidative stress drugs in treating schizophrenia (38, 39), and findings from animal models on oxidative stress and schizophrenia (40, 41). These highly cited articles laid foundations for research in the field and increased researcher awareness of the complex role oxidative stress may have in the pathology and treatment of schizophrenia.

Keyword co-occurrence analysis illustrates closeness in meaning and frequency of relevant research terms in a specific scientific field (42). In this study, five major research clusters emerged in the analysis of literature on schizophrenia and oxidative stress. In Cluster 1, “nitric oxide” appeared frequently. Nitric oxide is an intracellular and intercellular messenger in the brain; a reduction of nitric oxide activity appears to be involved in the pathogenesis of schizophrenia (43). Hence, in recent years, nitric oxide has been considered as a new possible target in the treatment of schizophrenia (44). Animal experiments have found the antioxidant N-acetyl-L-cysteine may restore behavioral deficits in a neurodevelopmental model of schizophrenia through a mechanism involving nitric oxide (45), and potentiators of nitrergic activity may alleviate symptoms of schizophrenia (44). In Cluster 2, non-schizophrenia psychiatric disorders associated with oxidative stress such as “bipolar disorder”, “depression” and “anxiety” were commonplace. Genome-wide analysis has shown a variety of psychiatric disorders share common risk genes and mutation sites (46). As well, transcriptome sequencing has suggested that schizophrenia and bipolar disorder have a similar interconnected pathway network centered on lysosomal function and the regulation of actin cytoskeleton (47). In Cluster 3, certain antipsychotics such as “haloperidol” and “clozapine” were popular search terms. Studies have found endocrine and cognitive side effects of certain traditional antipsychotic medications (e.g., haloperidol) in schizophrenia patients may be related to oxidative stress (48, 49). In contrast, the effect of second-generation antipsychotics on negative symptoms has been hypothesized to relate to antioxidant effects (50). In Cluster 4, “mitochondria” emerged as a keyword closely related to oxidative stress. Mitochondria-controlled neuropeptide release induces resistance to oxidative stress (51) and use of antioxidants could repair mitochondrial function in damaged central neurons represents a new direction in the treatment of various neuropsychiatric diseases (52). Cluster 5 appeared to reflect mediating influences on relations between schizophrenia and oxidative stress such as “polymorphism”. For instance, transferrin gene polymorphism is posited to be involved in mediating effects of psychotic symptoms on the relationship between oxidative stress and cognition in patients with chronic schizophrenia (53).

Burst detection analysis is used to illuminate the evolution of research trends in a given discipline. The keyword analysis with high citation bursts indicated that “tardive dyskinesia” related research was a popular focus in early studies between 1996 and 2011, perhaps because this is a severe side effect of antipsychotic drugs that is associated with oxidative stress and affects patient quality of life. Inflammation has been an ongoing burst keyword since 2016, and underscores an interest in disentangling complicated relations between “inflammation”, “oxidative stress” and “schizophrenia”. The “dual-hit” hypothesis of schizophrenia contends that an initial “hit” (e.g., genetic predisposition or prenatal insult) predisposes an individual susceptible to develop the disease, while a second “hit” (e.g., environmental adversity, or substance abuse) induces the full-blown syndrome; both of these hits could cause damage to the central nervous system through common pathways, including inflammation and oxidative stress (54). Recent research has suggested that the endocannabinoid system may be an intermediary pathway (55) that may become a promising new therapeutic target in the future. Overall, the research keyword analysis on schizophrenia and oxidative stress suggests there has been a transition from research focused on pathways to animal models, and subsequently to clinical applications.

This is the first bibliometric analysis to have explored the field of schizophrenia and oxidative stress-related research. Although this study helps to illustrate trends in research on oxidative stress and schizophrenia, its main limitations should be acknowledged. First, although WOS is a reliable, widely used bibliometric database, its use could have led to the omission of some studies, particularly from non-English language sources. Second, some research institutions have different names at different points in history, and such changes cannot be recognized in current bibliometric analysis approaches.

In conclusion, research on the association between oxidative stress and schizophrenia has received growing attention in the academic literature that is expected to continue its upward trajectory during the next two decades. To date, the United States has been the most productive region in generating associated research although international collaborations have contributed to the quantity and impact of studies in this field. In addition, the keyword analysis from this study suggested there has been a shift away from pathways between schizophrenia and oxidative stress to animal models, and, more recently, to clinical applications. As such, intervention studies on oxidative stress and schizophrenia are likely to be an important focus of related work in the near future.

## Data availability statement

The original contributions presented in the study are included in the article/Supplementary material, further inquiries can be directed to the corresponding authors.

## Author contributions

Study design: M-YC, X-HL, and Y-TX. Data collection, analysis, and interpretation: M-YC, QZ, Y-FL, W-YZ, TS, ZS, and TC. Drafting of the manuscript: M-YC and Y-TX. Critical revision of the manuscript: TJ. Approval of the final version for publication: all authors. All authors contributed to the article and approved the submitted version.
