# Supplementary material for: Schizophrenia and oxidative stress from the perspective of bibliometric analysis
Source: Front Psychiatry. 2023 Feb 27;14:1145409. doi: 10.3389/fpsyt.2023.1145409 (PMC10008861; doi:10.3389/fpsyt.2023.1145409)
Supplement: Supplementary file 1 [file Data_Sheet_1.docx]

Supplementary Material

Search terms.

Figure S1. International collaboration analysis and the ranking collaborations between countries (frequency > 30) based on schizophrenia and oxidative stress-related publications.

Figure S2. Overlay visualization map of worldwide institutions co-authorship analysis on schizophrenia and oxidative stress.

Figure S3. Overlay visualization map of author co-authorship analysis on schizophrenia and oxidative stress

Table S1. Top 10 journals with most publications in the field of schizophrenia and oxidative stress research.

Table S2. Top 10 articles with the most citations in the field of schizophrenia and oxidative stress research.

Search terms:

(TS= (“schizophren*” OR “Schizoaffective*”)) AND (TS= (“oxidative stress” OR “oxidant stress” OR “Oxidative Stresses” OR “Stress, Oxidative” OR “Antioxidative Stress” OR “Antioxidative Stresses” OR “Stress, Antioxidative” OR “Anti-oxidative Stress” OR “Anti oxidative Stress” OR “Anti-oxidative Stresses” OR “Stress, Anti-oxidative” OR “Oxidative Damage” OR “Damage, Oxidative” OR “Oxidative Damages” OR “Oxidative Stress Injury” OR “Injury, Oxidative Stress” OR “Oxidative Stress Injuries” OR “Stress Injury, Oxidative” OR “Oxidative Injury” OR “Injury, Oxidative” OR “Oxidative Injuries” OR “Oxidative Cleavage” OR “Cleavage, Oxidative” OR “Oxidative Cleavages” OR “Oxidative DNA Damage” OR “DNA Damage, Oxidative” OR “Damage, Oxidative DNA” OR “Oxidative DNA Damages” OR “DNA Oxidative Damage” OR “DNA Oxidative Damages” OR “Damage, DNA Oxidative” OR “Oxidative Damage, DNA” OR “Oxidative and Nitrosative Stress” OR “Oxidative Nitrative Stress” OR “Nitrative Stress, Oxidative” OR “Oxidative Nitrative Stresses” OR “Stress, Oxidative Nitrative” OR “Nitro-Oxidative Stress” OR “Nitro Oxidative Stress” OR “Nitro-Oxidative Stresses” OR “Stress, Nitro-Oxidative” OR “Stresses, Nitro-Oxidative” OR “protein carbonyl*” OR “glutathione” OR “reactive oxygen species” OR “free radicals” OR “nitric oxide” OR “lipid peroxidation” OR “malondialdehyde” OR “thiobarbituric acid reactive substances” OR “Antioxidant*”))

**
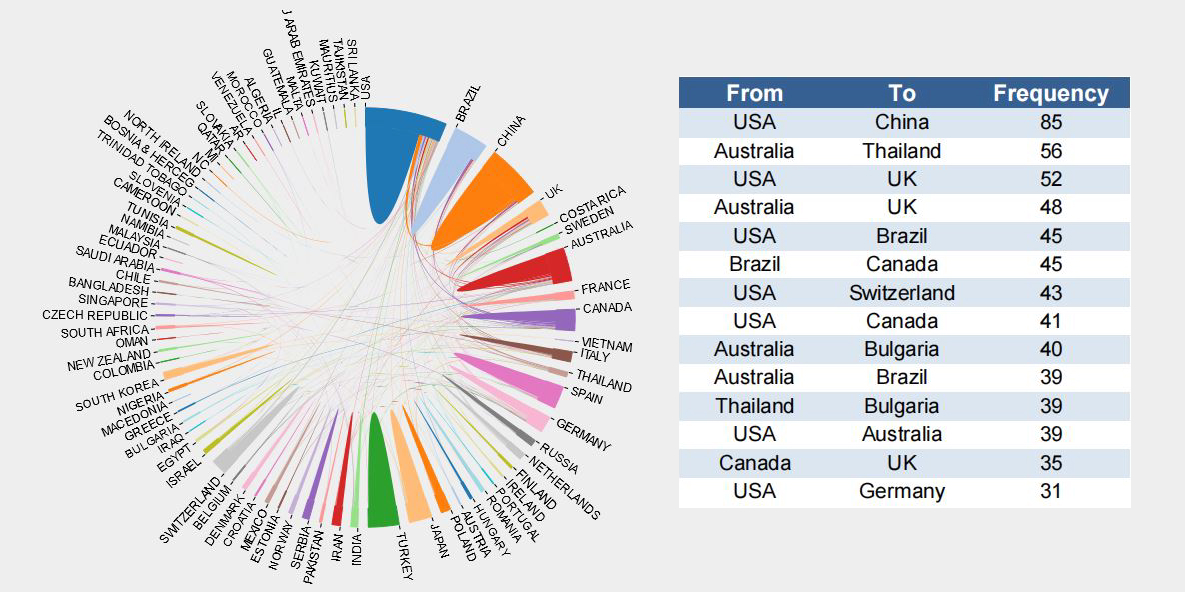
**

**Figure S1.** International collaboration analysis and the ranking collaborations between countries (frequency > 30) based on schizophrenia and oxidative stress-related publications.

**
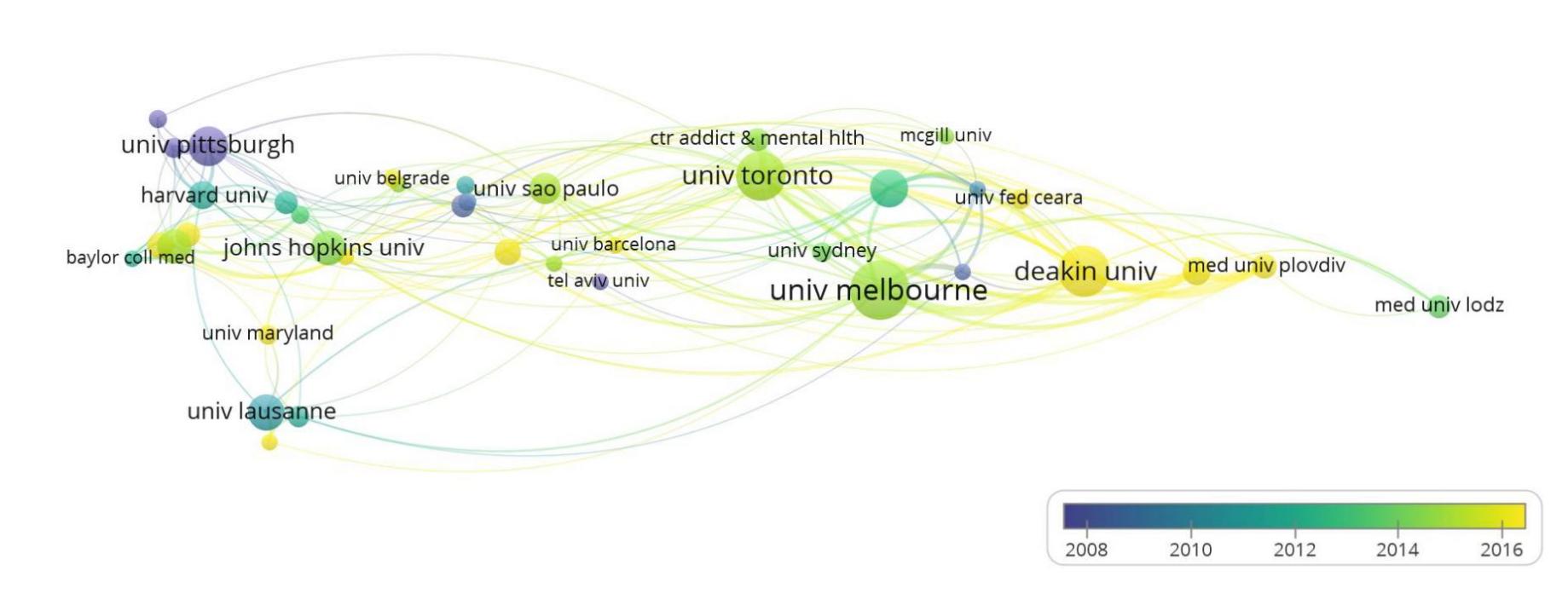
**

**Figure S2.** Overlay visualization map of worldwide institutions’ co-authorship analysis on schizophrenia and oxidative stress.

**
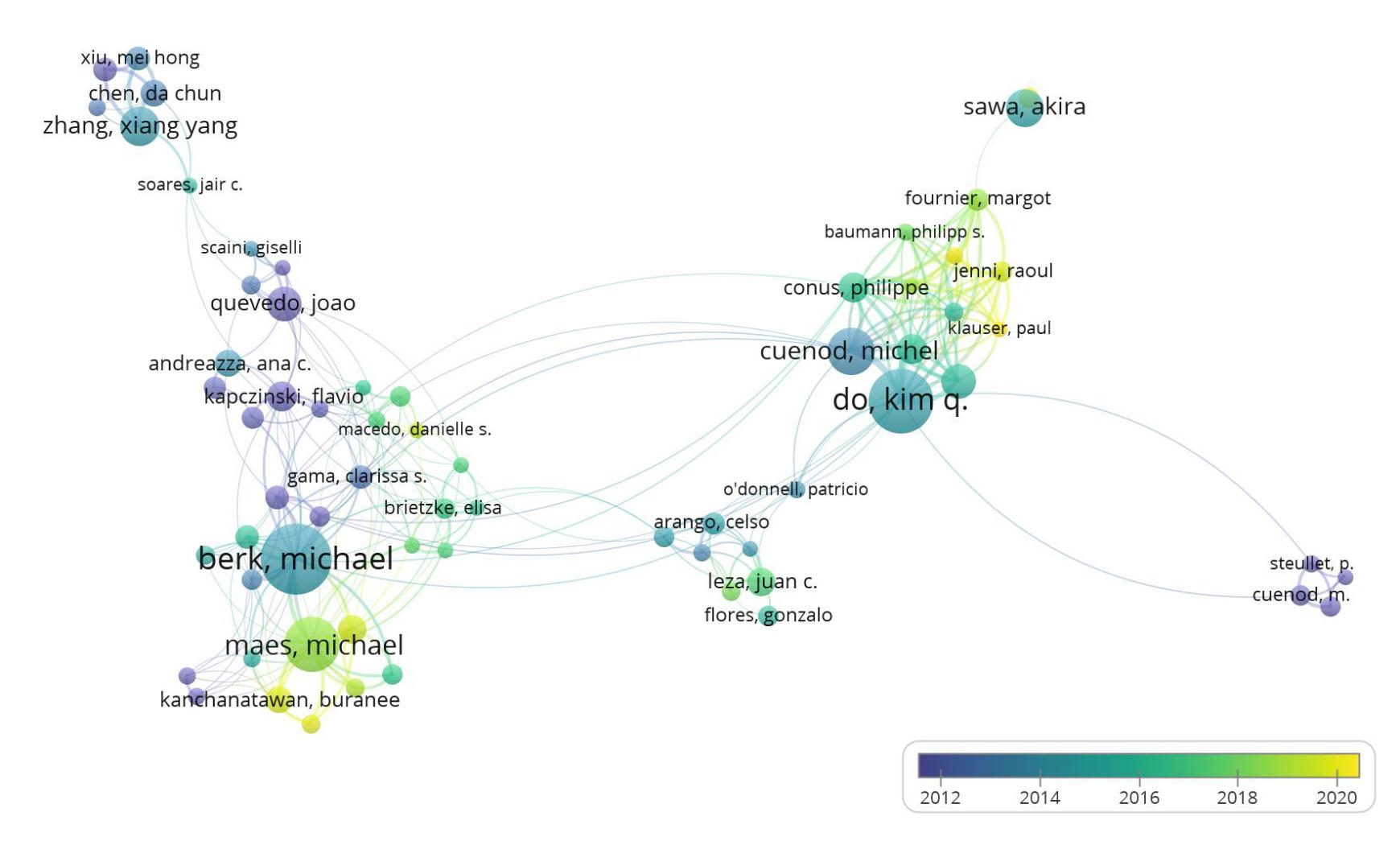
**

**Figure S3.** Overlay visualization map of authors’ co-authorship analysis on schizophrenia and oxidative stress.

**Table S1.** Top 10 journals with most publications in the field of schizophrenia and oxidative stress research.

| **Ranging** | **Journal** | **Article** | **Country** | **IF (2021)** | **JCR-c** |
| --- | --- | --- | --- | --- | --- |
| 1 | Schizophrenia Research | 172 | Netherlands | 4.662 | Q2 |
| 2 | Biological Psychiatry | 108 | USA | 12.810 | Q1 |
| 3 | Progress in Neuro-Psychopharmacology & Biological Psychiatry | 106 | UK | 5.201 | Q2 |
| 4 | Psychiatry Research | 80 | Netherlands | 11.225 | Q1 |
| 5 | Schizophrenia Bulletin | 68 | USA | 7.348 | Q1 |
| 6 | Journal of Psychiatric Research | 65 | UK | 5.250 | Q2 |
| 7 | European Neuropsychopharmacology | 63 | Netherlands | 5.415 | Q1 |
| 8 | Molecular Psychiatry | 55 | UK | 13.437 | Q1 |
| 9 | International Journal of Neuropsychopharmacology | 47 | UK | 5.678 | Q1 |
| 10 | Psychopharmacology | 47 | Germany | 4.415 | Q2 |

IF, impact factor (2020–2021); JCR-c, Journal Citation Reports category (2021)

**Table S2.** Top 10 articles with the most citations in the field of schizophrenia and oxidative stress research.

| **Year** | **Correspond-ing author** | **Corresponding author country** | **Journal** | **Title** | **Total citation** | **TC** | **TC/Y** |
| --- | --- | --- | --- | --- | --- | --- | --- |
| 2001 | Danbolt, NC | Norway | Progress in Neurobiology | Glutamate uptake | 3489 | 19.52 | 158.59 |
| 2001 | Swerdlow, NR | USA | Psychopharmacology | [Pharmacological studies of prepulse inhibition models of sensorimotor gating deficits in schizophrenia: a decade in review](https://www-webofscience-com.libezproxy.um.edu.mo/wos/woscc/full-record/WOS:000170351600002) | 1212 | 6.78 | 55.09 |
| 2004 | Bahn, S | UK | Molecular Psychiatry | [Mitochondrial dysfunction in schizophrenia: evidence for compromised brain metabolism and oxidative stress](https://www-webofscience-com.libezproxy.um.edu.mo/wos/woscc/full-record/WOS:000222257800006) | 754 | 14.34 | 39.68 |
| 2005 | Lieberman, JA | USA | Molecular Psychiatry | [Treatments for schizophrenia: a critical review of pharmacology and mechanisms of action of antipsychotic drugs](https://www-webofscience-com.libezproxy.um.edu.mo/wos/woscc/full-record/WOS:000225888300007) | 681 | 11.03 | 37.83 |
| 2008 | Bush, AI | Australia | International Journal of Neuropsychopharmacology | [Oxidative stress in psychiatric disorders: evidence base and therapeutic implications](https://www-webofscience-com.libezproxy.um.edu.mo/wos/woscc/full-record/WOS:000259706800010) | 615 | 8.81 | 41.00 |
| 1993 | Jones, EG | USA | Arch Gen Psychiatry | Altered distribution of nicotinamide-adenine dinucleotide phosphate-diaphorase cells in frontal lobe of schizophrenics implies disturbances of cortical development. | 559 | 4.88 | 18.63 |
| 2008 | Bogerts, B | Germany | [Journal of Psychiatric Research](https://www.sciencedirect.com/journal/journal-of-psychiatric-research) | [Immunological aspects in the neurobiology of suicide: Elevated microglial density in schizophrenia and depression is associated with suicide](https://www-webofscience-com.libezproxy.um.edu.mo/wos/woscc/full-record/WOS:000251558300008) | 518 | 7.42 | 34.53 |
| 1998 | Horrobin, D | Canada | Biological Psychiatry | [Depletion of omega-3 fatty acid levels in red blood cell membranes of depressive patients](https://www-webofscience-com.libezproxy.um.edu.mo/wos/woscc/full-record/WOS:000072260000002) | 459 | 5.97 | 18.36 |
| 2013 | Maes, M | Australia | BMC Medicine | [Aspirin: a review of its neurobiological properties and therapeutic potential for mental illness](https://www-webofscience-com.libezproxy.um.edu.mo/wos/woscc/full-record/WOS:000323166800001) | 446 | 7.81 | 44.60 |
| 2010 | Boksa, P | Canada | Brain, Behavior, and Immunity | [Effects of prenatal infection on brain development and behavior: A review of findings from animal models](https://www-webofscience-com.libezproxy.um.edu.mo/wos/woscc/full-record/WOS:000280029500005) | 429 | 9.36 | 33.00 |
